# IUC24400-75 Real-world outcomes of second-line pembrolizumab in urothelial cancer: Campania network analysis

**DOI:** 10.1093/oncolo/oyaf248.022

**Published:** 2025-08-29

**Authors:** R Tambaro, E Perri, A Muto, L Formisano, G Di Lorenzo, C Buonerba, D Bosso, F Sabbatino, E Coppola, S Pignata

**Affiliations:** Uro-Gynecological Department, Istituto Nazionale Tumori di Napoli, IRCCS “G. Pascale”, Naples, Italy; Departement of Precision Medicine, Università degli Studi della Campania “L.Vanvitelli”, Naples, Italy,; Division of Medical Oncology, “San Giuseppe Moscati” Hospital, Avellino, Italy; Department of Clinical Medicine and Surgery, University of Naples “Federico II”, Napoli, Italy,; Oncology Unit, “Andrea Tortora” Hospital, Pagani, Italy,; Oncology Unit, “Andrea Tortora” Hospital, Pagani, Italy,; Oncology Unit, Ospedale del Mare, Naples, Italy,; Oncology Department, University Hospital “San Giovanni di Dio e Ruggi d‘Aragona”, Salerno, Italy; Uro-Gynecological Department, Istituto Nazionale Tumori di Napoli, IRCCS “G. Pascale”, Naples, Italy; Uro-Gynecological Department, Istituto Nazionale Tumori di Napoli, IRCCS “G. Pascale”, Naples, Italy

## Abstract

**Background:**

Platinum-based chemotherapy followed by immunotherapy is standard for locally advanced/metastatic urothelial cancer (La/mUC). Despite advances, La/mUC remains aggressive with poor prognosis. Real-world outcomes may differ from clinical trials. Oncology networks generating real-world data (RWD) are crucial to guide treatment. We retrospectively analyzed La/mUC patients treated with second-line pembrolizumab within the Campania Oncological Network (ROC).

**Methods:**

This multicenter retrospective study included patients (≥18 years) with histologically/cytologically confirmed La/mUC, previously treated with chemotherapy, who received pembrolizumab (200 mg every 3 weeks) across 6 ROC centers. Primary endpoints were progression-free survival (PFS) and overall survival (OS); secondary endpoints included objective response rate (ORR), disease control rate (DCR), and safety.

**Results:**

From January 2021 to November 2023, 132 patients received pembrolizumab. Median age was 67 years (range 30-88); 73.5% were male. Eastern Cooperative Oncology Group (ECOG) performance status was 0-1 in 82.6%. Most had pure urothelial carcinoma (87.1%), and 12.9% had rare variants. At diagnosis, 34.1% had metastatic disease, and 54.5% underwent cystectomy. Common metastatic sites were lymph nodes (74.2%), lung (38.6%), bone (34.1%), and liver (15.9%). After a median follow-up of 8.5 months, 81.8% experienced progression or death. Median PFS and OS were 3.75 (95% CI, 3.4- 4.7) and 7.3 (95% CI, 6.05-9.33) months, respectively. ORR was 13.5% (95% CI, 7.4%-19.7%), DCR 33.9% (95% CI, 25.6%-42.0%), and ORR in rare subtypes was 23.5% (95% CI, 3.3%-43.7%).

Metastatic disease at diagnosis (HR = 2.01, *P* = .02) and liver metastases (HR = 2.11, *P* = .02) were associated with worse OS. Lymph node-only metastases predicted better PFS (HR = 0.46, *P* = .004) and OS (HR = 0.52, *P* = .02). Prior cystectomy improved PFS (HR = 0.67, *P* = .04) and OS (HR = 0.54, *P* = .003) in univariate analysis.

Among 114 treatment-related adverse events (AEs), 79.8% were grade 1-2, and 20.2% grade 3-4. No treatment-related deaths occurred. Pembrolizumab was discontinued in 7.6% due to AEs; the most frequent were asthenia (20%), pruritus (10%), and myalgia (7%).

**Conclusions:**

Second-line pembrolizumab showed clinical activity and acceptable safety in this real-world La/mUC cohort. Prognostic factors such as metastatic sites and prior cystectomy significantly influenced outcomes. These data support the role of oncology networks in guiding real-world treatment strategies.

